# Adverse events related with propylthiouracil therapy of Graves’ Disease in children at Cipto Mangunkusumo Hospital Jakarta

**DOI:** 10.1186/1687-9856-2013-S1-P139

**Published:** 2013-10-03

**Authors:** Novina Andriana, Aman B Pulungan, Bambang Tridjaja

**Affiliations:** 1 Division of Pediatric Endocrinology, Department of Pediatrics, Faculty of Medicine, Padjadjaran University, Bandung, Indonesia; 2 Division of Pediatric Endocrinology, Department of Pediatrics, Faculty of Medicine, University of Indonesia, Jakarta, Indonesia

## 

Despite propylthiouracil (PTU) and methimazole (MMI) having been used for more than a half century to treat hyperthyroidism caused by Graves’ disease, controversy still exists in antithyroid drug (ATD) therapy because of their significant side effects. Reports of adverse events involving cutaneous reactions, vasculitis, agranulocytosis and hepatotoxicity have appeared. In Indonesia, PTU was still included in National Essential Drugs List to treat Graves’ disease in children. The aim of our study was to provide insights into adverse events that can be associated with ATD use. We reviewed adverse events associated with ATD therapy in pediatric patients with Graves’ disease from September 2009 to June 2012 at outpatient clinic, Cipto Mangunkusumo Hospital, Jakarta. We found 21 patients with Graves’ disease aged 3-to16 yr old, 5 male and 16 female. During this periode, 9 patients (43%) treated with an average daily dose of PTU, the rest with MMI (57%). Adverse events attributed to PTU use were seen in 2 patients. Liver dysfunction was observed in 1 patient, alanine aminotransferase, aspartate aminotransferase and blood bilirubin were increased. The second one, developed epistaxis and had 2 times relaps episodes of thyrotoxicosis and suggested to perform thyroidectomy procedure. Four of the patients treated with PTU switched to MMI because of increased ALT/AST. None of them required hospitalization. One patient treated with PTU had full remission after 5 years of therapy. There was no adverse event observed in patients treated with MMI. Our results suggested that PTU use in children need routine biochemical monitoring to prevent severe adverse events.

